# Preclinical Models: Boosting Synergies for Improved Translation

**DOI:** 10.3390/jcm9041011

**Published:** 2020-04-03

**Authors:** Chiara Attanasio, Mara Sangiovanni

**Affiliations:** 1 Department of Veterinary Medicine and Animal Productions, University of Naples Federico II, 80137 Napoli, Italy; 2 Center for Advanced Biomaterials for Health Care—Istituto Italiano di Tecnologia, 80125 Napoli, Italy; 3 Interdepartmental Center for Research in Biomaterials (CRIB) University of Naples Federico II, 80125 Napoli, Italy; 4 Stazione Zoologica “Anton Dohrn”, 80122 Napoli, Italy; mara.sangiovanni@szn.it

The field of preclinical models is a very vast arena, in which finding connections among groups acting in apparently very distant research areas can sometimes prove challenging. An osmosis of mindset and competencies (methodologies, techniques, models), along with a comparison of different standpoints, is always an opportunity to reflect on where one’s work stands in a wider scenario. The goal of this Special Issue is to collect information, and ultimately share ideas and foster debate about different approaches to translational model research. The eleven papers composing this issue will be of interest to researchers looking for an update of the currently heterogeneous panorama of preclinical models and to those in search of inspiring ideas in the field.

Figure 1 shows a visual representation of the connections among the papers here presented. Each colored ribbon relates a paper with a topic. It is immediately apparent how many topics are addressed and how many relationships exist among the different research areas. Multidisciplinarity, which is intrinsic to the very nature of preclinical models, emerges at first sight as a relevant feature.

Out of eleven papers, six are based on animal models [1,2,3,4,5,6], highlighting that these models still play a crucial role in translational medicine, even in a historical moment in which the need to find alternative methodologies is increasingly pressing.

In this context, the issue raised by some authors about the translational validity of a certain animal model, in this specific case the Reeler mouse, is very timely. Central to the debate is the rare occurrence of the very conditions for which mice homozygous for the Reeler mutation have been created, and the objective difficulty of fully validating the mice expressing the heterozygous genotype as a translational model for more frequent diseases such as autism and schizophrenia [2].

After all, animal models are often suspended “halfway” between being widely accepted as good tools for basic research and being recognized for their translational potential. Therefore, this issue should always be considered when somebody, whether experimenter or modeler, decides to work with them. In this regard, one paper focuses on the improvement of cellular and animal models of chondrosarcoma. The authors [4] provide four cell lines, displaying tumorigenic and invasive features suitable to be used as valuable alternatives to veteran endless passaged cell lines. They also detail the genetic drift that these cells underwent as an adaptive response to in vitro and in vivo expansion.

Cancer, and more specifically the usage of nanoparticles (NP) both for multimodal imaging and as contrast agents (CAs), is the topic of a review [8] which highlights the advantages of using NP-based PET/MRI multimodal imaging in tumor diagnosis and characterization. Additionally, these nanosystems can be applied to theranostics in the very prominent scenario of personalized medicine. The authors point to multidisciplinarity as an essential requisite to deeply understand the possible applications and the underlying biomolecular processes of the targeted diseases.

The centrality of interdisciplinary synergies is further highlighted in a paper [6] specifically focused on the high potential held by MRI imaging in translational oncology. By analyzing several disease models and cancer types, the authors present their approach to reduce the gap from preclinical applications to clinical practice.

The “from bench to bedside” path is also the leitmotif of an article [9] addressing several issues related to the use of human-induced pluripotent stem cells (iPSC). More specifically, the paper analyses the potential of iPSC-endothelial cells in accelerating tissue regeneration and their suitability to enter progressively more clinical trials. The most represented research field in this Special Issue is tissue morphology [1,2,3,5] followed by tissue engineering [9,10,11] and neuroscience [1,2,3], with one article intercepting both these areas of expertise [12]. This latter work reports, indeed, a method to generate a three-dimensional neuronal system composed of cortical neurons and glial cells derived from iPSCs, suitable for drug screening and disease modeling.

Good news from the field of skin regeneration: the work on bioengineered skin substitutes has been selected by the editorial board as the cover story of the issue of December 2019 (www.mdpi.com/2077-0383/8/12). The article, presented by a group active in the field of dermal substitutes [13] and three-dimensional tissue-like models [14], is focused on the analysis of the most modern strategies to overcome the scarring process and promote skin regeneration, by implanting an engineered dermis capable of recapitulating the architecture and presenting molecular signals similarly to the native dermis [10].

The essential role of tissue-engineered constructs in mimicking extra-cellular matrix morphology and function is also highlighted in another work, in which the authors analyze the most promising fabrication technologies in the field. In particular, the paper reports the real effectiveness of the “bottom-up” approach for cell-free and cell-laden scaffolds in tissue and organ bioengineering [11].

Passing to the fascinating and complex field of neuroscience, two original research articles contributed to this Special Issue. It is worth noting how they both witness the increasing value of the short-lived African turquoise killifish *Nothobranchius furzeri* as an emerging vertebrate model in aging research. In particular, one paper describes, for the first time, the expression of neurotrophin-6 in different brain areas in both young and old animals [1], while the other one provides the first evidence of nucleobindin-2/nesfatin-1 expression and its role as a food intake regulator in vertebrate aging [3].

Finally, very high translational potential arises from a work proving the effectiveness of human red blood cells to act as oxygen carriers for graft preservation in liver transplantation. In particular, the study is aimed at enhancing the potential of normothermic machine perfusion, a modern methodology applied to organ preservation [15]. In view of the crucial role of transplantation for patients with end-stage disease and the consequent increasing demand for the inclusion of marginal donors, new methods to improve organ preservation and, eventually, induce graft repair are undoubtedly relevant to the clinical setting [5].

An important contribution might come from *in silico* modeling, a field that has unexpectedly been neglected in this Special Issue despite being a constitutive piece of the puzzle. In fact, *in silico* models prove to be useful at several stages of the process from research to clinical application, in a vision that aims at a deep digital transformation of all the production steps. This might be accomplished either with data-driven approaches, such as in omics or materiomics simulations, or with mechanistic modeling (e.g., bioreactors process) in tissue engineering [16]. Another interesting avenue is the development of *in silico* models capable of exploiting patient-specific data to build personalized medical treatments, such as three-dimensional mathematical models of tumors [17]. The urgency of an integrated approach merging *in vivo* experiments and *in silico* representations to obtain more powerful descriptive and predictive models is also emerging: for instance, the integration of microfluidic devices and computational modeling to better study vascularization dynamics in cancer [18]. 

The harmonization of data coming from different fields [19,20] and the exchange of expertise at several levels are fundamental parts of an essential strategy whose final aim is to accelerate the translation and the design of more precise preclinical models, in a true accomplishment of the “from bench to bedside” paradigm.

## Figures and Tables

**Figure 1:**
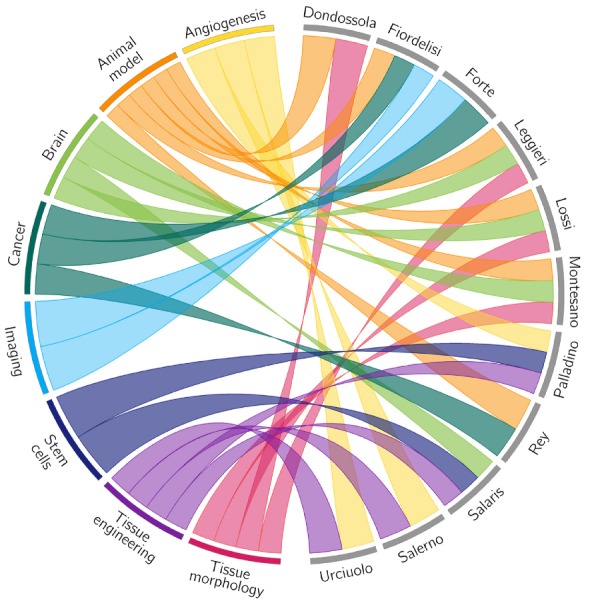
A visual representation of the connections among the papers presented in this Special Issue. Several topics were defined by widening the keywords specified in each paper (colored sectors on the left side of the image). Papers (gray sectors on the right side of the image) are identified by the first author’s name. Colored ribbons connect papers with the topics treated. The figure was made with the Circos software [7].
